# 

**Published:** 1872-09

**Authors:** 


Vol. XII.] SEPTEMBER, 1872. [No. 2.

### BUFFALO

### Medical and Surgical Journal.

EDITED BY JULIUS F. MINER, M. D.

Professor of Special Surgery in the Buffalo Medical College ; Surgeon to the Buffalo General Hospital ; Surgeon to Buffalo Hospital of the Sisters of Charity, etc., etc.

BUFFALO.

PUBLISHED FOR THE EDITOR.

1872.

